# Supplementary material for: TOC1–PIF4 interaction mediates the circadian gating of thermoresponsive growth in Arabidopsis
Source: Nat Commun. 2016 Dec 14;7:13692. doi: 10.1038/ncomms13692 (PMC5171658; doi:10.1038/ncomms13692)
Supplement: Supplementary Information — Supplementary Figures 1 - 10 and Supplementary Table 1 [file ncomms13692-s1.pdf]

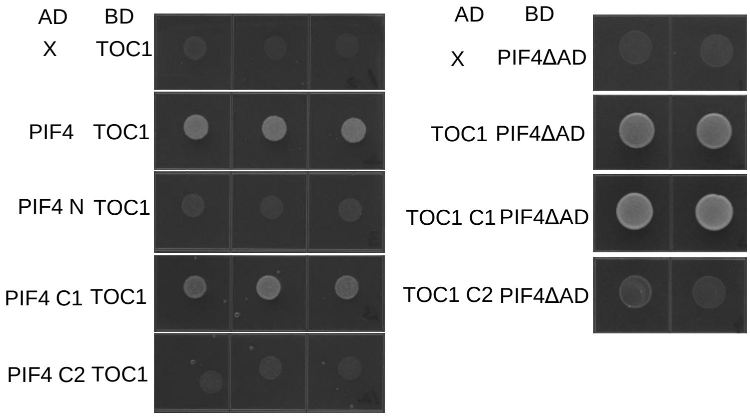

Supplementary Figure 1: Yeast two-hybrid assays showing the interaction between PIF4 and TOC1

The yeast clones transformed with the indicated constructs were grown on synthetic dropout medium without histidine plus 1 mM 3AT. Information on the various fragments of PIF4 and TOC1 is shown in Fig. 1a and 1b.

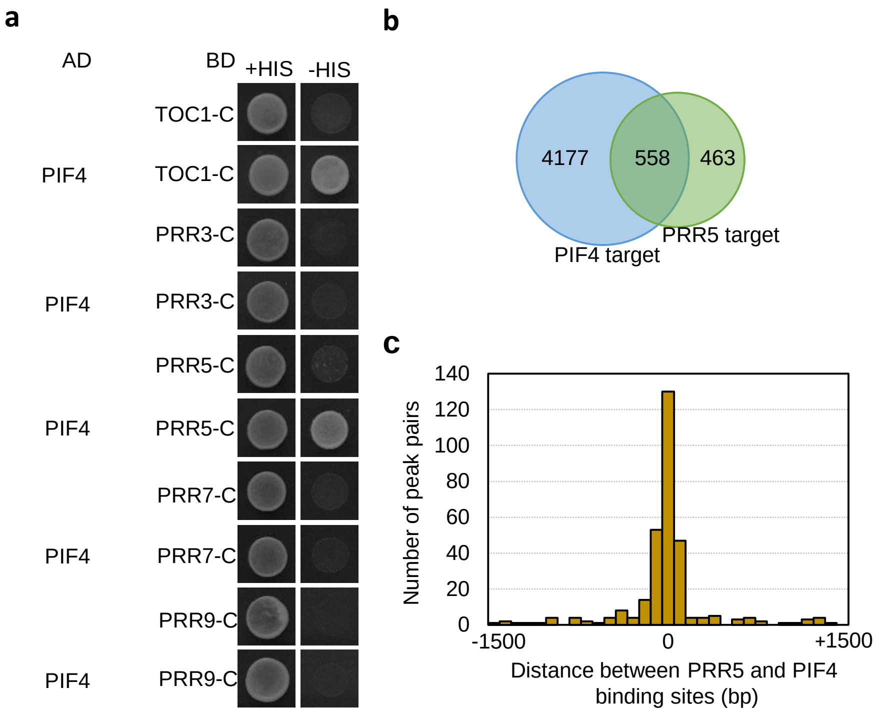

Supplementary Figure 2: PRR5 directly interacts with PIF4
(a) Yeast two-hybrid assays. The yeast clones transformed with the indicated constructs were grown on the synthetic dropout medium (+HIS) or synthetic dropout medium without histidine plus 5 mM 3AT (-HIS). The C-terminal domains of TOC1 and PRRs (TOC1-C: amino acid 325-618; PRR3-C: amino acid 264 to 522; PRR5-C: amino acid 299-558; PRR7-C: amino acid 328 -727; PRR9-C: amino acid 263-468) were used in the yeast two-hybrid assays to avoid self-activation of BD-fusion proteins.
(b) Venn diagram shows the overlap between PIF4 and PRR5 target genes that were identified in the previous ChIP-Seq assays (Nakamichi et al., 2012; Oh et al., 2012). The overlap between PIF4 and PRR5 target genes is statistically significant (Fisher's exact test $p < 2*10^{-16}$).
(c) Distribution of distances between binding sites of PRR5 and PIF4 in their common target genes identified in (b).

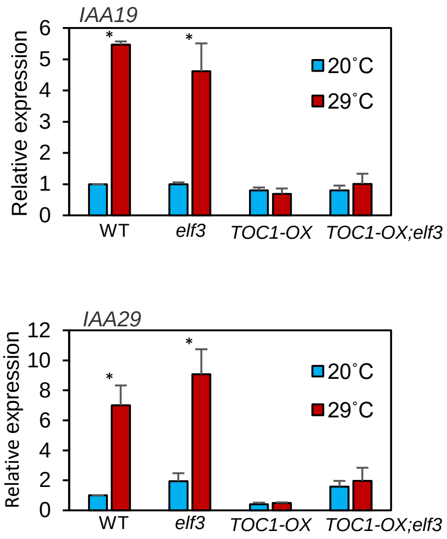

Supplementary Figure 3: TOC1 suppresses the warm temperature activation of *IAA19* and *IAA29* expression independent of ELF3.

Seedlings were entrained in light/dark cycles (12L:12D) at 20˚C for 4 days. On the 5th day, the seedlings were treated with warm temperature (29˚C) or kept at 20˚C for 8 hours at ZT0, then harvested for RNA extraction at ZT8. The gene expression levels were normalized to *PP2A* and presented as values relative to that of wild type at ZT0. Error bars indicate s.d. (*n*=3). * $p < 0.05$ (Student's t-test).

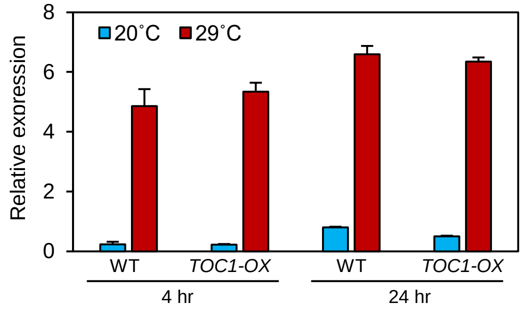

Supplementary Figure 4: *HSP70* expression is induced by warm temperature in *TOC1-OX*

Seedlings grown at 20˚C for 5 days were incubated at 20˚C or 29˚C for 4 hours or 24 hours. The *HSP70* expression levels were normalized to that of WT at 20˚C (4 hr). Error bars indicate s.d. (*n*=3).

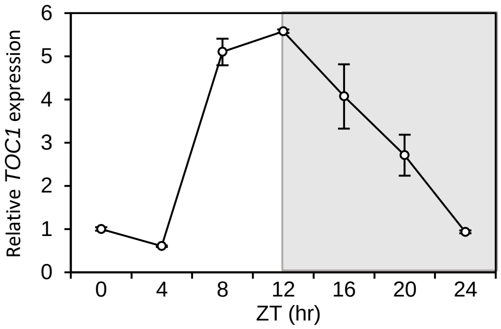

Supplementary Figure 5: *TOC1* expression under continuous white light after entrainment in light/dark cycles

Wild type seedlings were grown in light/dark cycles (12L:12D) at 20˚C for 4 days. On 5th day, the seedlings were transferred under the continuous light and harvested every 4 hours for RNA extraction. The expression levels of *TOC1* were normalized to *PP2A* and presented as values relative to that in ZT0. Shaded area indicates the subjective night. Error bars indicate S.D. (*n*=3).

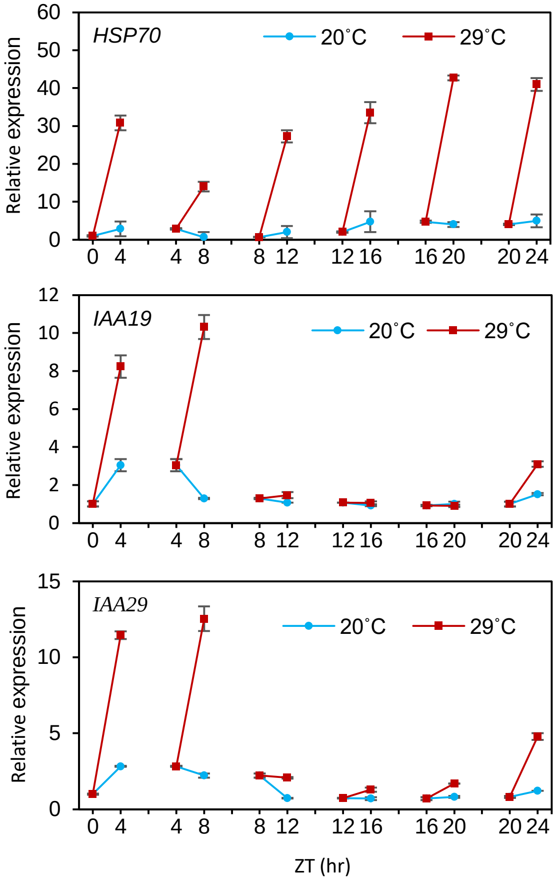

Supplementary Figure 6: qRT-PCR analysis of *HSP70*, *IAA19* and *IAA29* expression

Wild type seedlings were entrained in light/dark cycles (12L:12D) at 20˚C for 4 days and then transferred under the continuous light. At different ZTs, the seedlings were treated with warm temperature (29˚C) for 4 hours. The gene expression levels were normalized to *PP2A* and presented as values relative to that of wild type at ZT0. Error bars indicate s.d. (*n*=3).

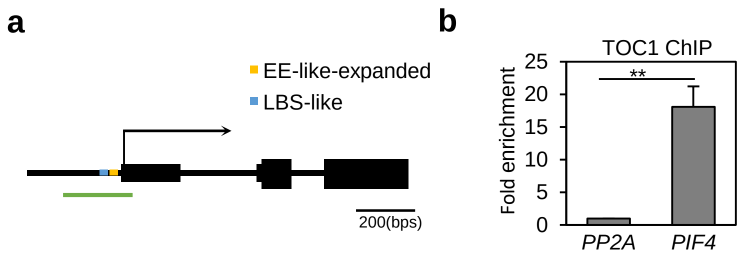

Supplementary Figure 7: TOC1 directly binds to the *PIF4* promoter

(a) Summary of the structure of the *PIF4* promoter. Green bar indicates position of qPCR amplicons for ChIP assay. LBS-like : LUX-Binding Site-like sequence.

(b) ChIP-qPCR assays of TOC1 binding on the *PIF4* promoter. Five-day-old *TOC1p::TOC1-YFP* seedlings were used for ChIP assay using anti-GFP antibody. The enrichment of DNA was calculated as the ratio between *TOC1p::TOC1-YFP* and wild type control, normalized to that of the *PP2A* coding region as an internal control. Error bars indicate s.d. (n=3). ** $p < 0.01$ (Student's t-test).

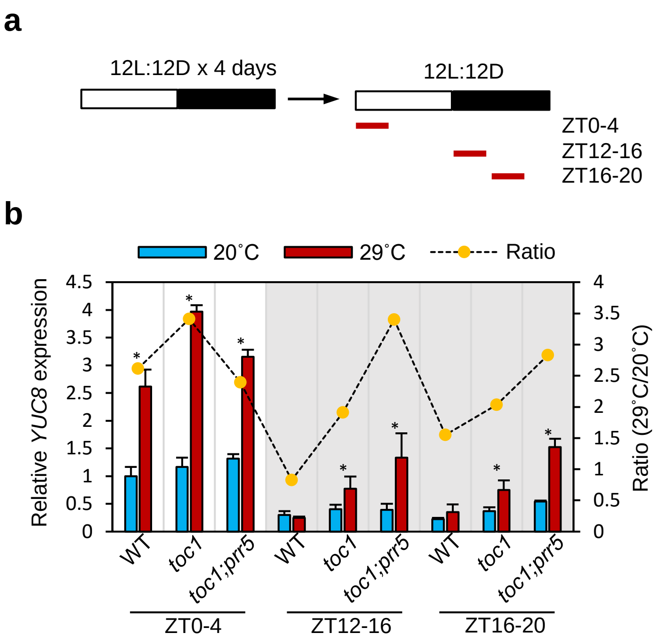

Supplementary Figure 8: The thermosensitivity of *YUC8* expression in the night of normal light/dark cycle is restored in *toc1;prr5* mutant

(a) Wild type seedlings were grown in light/dark cycles (12L:12D) at 20˚C for 4 days. On the 5th day, the seedlings were treated with warm temperature (29˚C) for 4 hours at different ZTs (ZT0-4, ZT12-16, and ZT16-20) and then harvested at ZT4, ZT16 and ZT20 for RNA extraction.

(b) Expression levels of *YUC8* were normalized to *PP2A* and presented as values relative to that of wild type at ZT0. Gray dots indicate the ratio of *YUC8* expression levels in the warm temperature-treated seedlings to non-treated seedlings. Shade areas indicate nights. Error bars indicate s.d. (n=3). * $p < 0.05$ (Student's t-test).

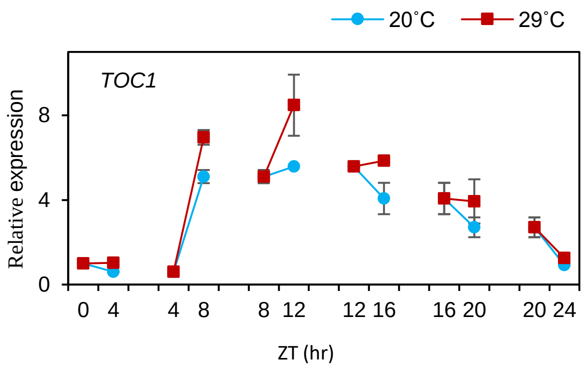

Supplementary Figure 9: qRT-PCR analysis of *TOC1* expression

Wild type seedlings were entrained in light/dark cycles (12L:12D) at 20˚C for 4 days and then transferred under the continuous light. At different ZTs, the seedlings were treated with warm temperature (29˚C) for 4 hours. The gene expression levels were normalized to *PP2A* and presented as values relative to that of wild type at ZT0. Error bars indicate s.d. (*n*=3).

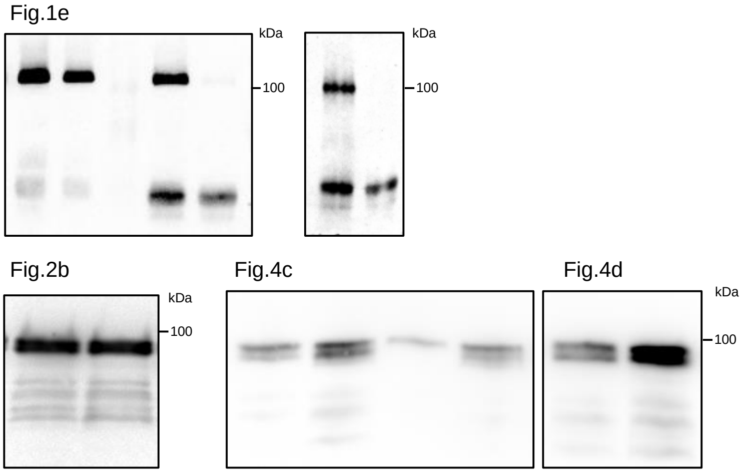

Supplementary Figure 10: The uncropped versions of gel images

Supplementary Table 1. Primer list for qRT-PCR and ChIP-PCR assays

qRT-PCR		
Gene	Forward	Reverse
PP2A	TATCGGATGACGATTCTTCGTGCAG	GCTTGGTCGACTATCGGAATGAGAG
PIF4	GCCAAAACCCGGTACAAAACCA	CGCCGGTGAACTAAATCTCAACATC
TOC1	TGCTGAGGTACATCACACGAGACAA	GTGCGAAGAGGCTTCACAAGGTAGT
YUC8	AAACGCTCAAGGGGTTCTCTTCG	CACGCACAACACCCTTTGATTCG
IAA19	GGTGACAACTGCGAATACGTTACCA	CCCGGTAGCATCCGATCTTTTCA
IAA29	AAACAGCGTTTGTTTGCCTTGAATG	TGGCCATCCAACAACTTCGCTAT
HSP70	GGGCACGAACAAAGGACAACAAC	CCTCAGCCGACACATTCAGGATAC
ChIP-PCR		
Gene	Forward	Reverse
PP2A	CGGCTTTCATGATTCCCTCT	GCCTTAAGCTCCGTTTCCTACTT
UBC30	CAAATCCAAAACCCTAGAAACCGAA	AACGACGAAGATCAAGAACTGGGAA
PIF4	CACTGATTCCAACACAATGTCC	GGTACAGACAGAAAGTGACAGGAG
IAA19	GTCTCCCCACACAAACTGAATAAC	CGTCGTGCTTTTTATATGTTGCTT
IAA29	GCCATATGGATATGGTCCTTCAAC	GAAATATCAACGTGAATGTCACGTG
YUC8	TGGTTCCACACAATTTTCACAG	GCAACGATGGTGATTGTTGAAG
